# The Relationship between Autism Spectrum Disorder and Melatonin during Fetal Development

**DOI:** 10.3390/molecules23010198

**Published:** 2018-01-18

**Authors:** Yunho Jin, Jeonghyun Choi, Jinyoung Won, Yonggeun Hong

**Affiliations:** 1 Department of Rehabilitation Science, Graduate School of Inje University, Gimhae 50834, Korea; jynh33@naver.com (Y.J.); yiopiop0011@nate.com (J.C.); 2 Ubiquitous Healthcare & Anti-aging Research Center (u-HARC), Inje University, Gimhae 50834, Korea; wy11167@naver.com; 3 Biohealth Products Research Center (BPRC), Inje University, Gimhae 50834, Korea; 4 Department of Physical Therapy, College of Healthcare Medical Science & Engineering, Inje University, Gimhae 50834, Korea

**Keywords:** autism spectrum disorder, melatonin, fetal development, neuroprotection, circadian rhythm

## Abstract

The aim of this review is to clarify the interrelationship between melatonin and autism spectrum disorder (ASD) during fetal development. ASD refers to a diverse range of neurodevelopmental disorders characterized by social deficits, impaired communication, and stereotyped or repetitive behaviors. Melatonin, which is secreted by the pineal gland, has well-established neuroprotective and circadian entraining effects. During pregnancy, the hormone crosses the placenta into the fetal circulation and transmits photoperiodic information to the fetus allowing the establishment of normal sleep patterns and circadian rhythms that are essential for normal neurodevelopment. Melatonin synthesis is frequently impaired in patients with ASD. The hormone reduces oxidative stress, which is harmful to the central nervous system. Therefore, the neuroprotective and circadian entraining roles of melatonin may reduce the risk of neurodevelopmental disorders such as ASD.

## 1. Introduction

Melatonin (*N*-acetyl-5-methoxytryptamine), a circadian rhythm-dependently synthesized and secreted hormone [1], was first structurally identified in 1958 [2]. Typically, melatonin levels peak at 60–200 pg/mL between 2 and 4 a.m. and decrease to 0–20 pg/mL during the day [3]. This hormone is produced mainly by the pineal gland, and other organs including the retina, Harderian gland, gut, bone marrow, platelets, glial cells, lymphocytes, pancreas, kidneys, and skin are also involved in the production of melatonin [4]. Melatonin is synthesized from its precursor, tryptophan, which becomes 5-hydroxytryptophan in a reaction catalyzed tryptophan hydroxylase [4]. Aromatic amino acid decarboxylase (AAD) converts 5-hydroxytryptophan into serotonin [4], which is then converted to *N*-acetylserotonin (NAS) by arylalkylamine *N*-acetyltransferase (AANAT) [4]. This acetylated form of serotonin, *N*-acetylserotonin is converted to melatonin through the action of hydroxyindole O-methyltransferase (HIOMT) [4]. Melatonin is considered to have various biological functions, including the regulation of circadian rhythm [5] and sleep [6], anti-cancer [7], metabolic effects [8], anti-inflammatory functions [9], and antioxidant effects [10,11]. Melatonin also plays a crucial role in fetal development. Because the pineal gland undergoes maturation after birth, the fetus is dependent on maternal melatonin. Melatonin can cross physiological barriers, including the blood-placenta barrier, without denaturation, and subsequently influences placental function [12]. During pregnancy, melatonin crosses the placenta and enters the fetal circulation, conveying photoperiodic information to the fetus. Consequently, melatonin affects the circadian rhythm of the offspring [13]. Several factors have been shown to alter circadian rhythms including jet lag [14], shift work [14], and melatonin levels [15]. Abnormal melatonin secretion has been implicated in circadian disturbances [16] and neurodevelopmental abnormalities including autism spectrum disorder (ASD) [17,18,19,20]. The antioxidant and direct free radical scavenging activities of melatonin are well known [21]. A previous study found that serum levels of melatonin were significantly higher in rats fed walnuts containing melatonin (38.0 ± 4.3 pg/mL) than in those fed a normal diet (11.5 ± 1.9 pg/mL). The higher melatonin levels in the walnut-fed group were linked to increased antioxidative capacity [22]. These findings are supported by a study in human, which found that the nighttime increase serum melatonin was associated with antioxidative activity of the hormone [23]. Moreover, previous findings suggest that the decrease susceptibility to oxidative stress and age-related degenerative diseases [23]. The relationship between melatonin and antioxidant activity has been investigated in other body fluids. The daytime melatonin levels in the bile of mammals (i.e., human, monkey, rat, guinea pig, rabbit, and pig) range from 2000 to 11,000 pg/mL, which is about three orders of magnitude higher than the daytime levels of serum melatonin. Bile acids and oxidized cholesterol derivatives may induce oxidative stress in the small intestine; therefore, high levels of melatonin in the bile are essential to prevent oxidative damage [24]. The direct free radical scavenging activity of melatonin, which reduces neuronal damage induced by oxidative stress, may decrease risk of neurodegenerative diseases [25].

Melatonin may play a role in neural development. A normal sleep pattern is essential for optimal neurodevelopment, and disrupted circadian rhythms, possibly due to abnormal melatonin concentrations, may diminish brain growth and increase the risk of ASD [13,26]. ASD is associated with low melatonin levels and sleep disorders, and individuals with ASD often have sleep problems, including going to bed, falling asleep, and prolonged sleep latency [27]. The administration of melatonin before sleep improves sleep efficiency in patients with ASD [28]. About two-thirds of children with ASD have chronic sleep disorders [29]. Moreover, the prevalence of sleep problems in children with ASD is 50–80% compared with 9–50% in normal children [30]. The causes of sleep disturbances in ASD are multifactorial, and although no single intervention is completely effective, melatonin is one of the most effective treatments for individuals with ASD [31]. High serum levels of serotonin, which can be converted into melatonin, are frequently observed in patients with ASD [32]. Moreover, ASD is thought to be closely associated with the serotonin-*N*-acetylserotonin (NAS)-melatonin pathway, which is often disrupted in individuals with ASD [33]. Recent findings suggest that the low melatonin levels in patients with ASD may be the result of reduced arylalkylamine I-acetyltransferase (AANAT) and hydroxyindole-O-methyltransferase enzyme activity during melatonin synthesis in the pineal gland, gut, and platelets [34]. NAS and *N*-acetylserotonin methyltransferase are highly heritable, with heritabilities of 0.72 ± 0.091 and 0.59 ± 0.097, respectively, compared with serotonin (0.31 ± 0.078), AANAT (0.34 ± 0.077), and melatonin (0.22 ± 0.071), suggesting that variations in melatonin synthesis in individuals with ASD are inherited [35]. Furthermore, low parental melatonin levels may increase the risk of ASD in offspring, underscoring the importance of melatonin during fetal neurodevelopment [36]. In a recent review of prevention and therapeutic strategies for patients with fragile X syndrome (FXS) and ASD, we reported a correlation between melatonin and ASD [37]. The focus of the current review is to elucidate the effect of melatonin on fetal neurodevelopment and the risk of ASD, which the aim of clarifying the relationship between melatonin and ASD during fetal development.

## 2. Melatonin and Its Regulatory Effects on Circadian Rhythm

### 2.1. Melatonin and Its Putative Role in Regulating Fetal Circadian Rhythm

Circadian rhythm refers to the fluctuation in the internal environment in living creatures depending on a 24 h daily cycle [38]. Mammalian daily rhythms are mainly regulated by the circadian master clock, the suprachiasmatic nucleus (SCN), which is located in the anterior hypothalamus [39]. This master clock has numerous clock cells that synchronize the 24 h of biological clock [40]. In turn, peripheral oscillators in other brain areas and peripheral organs initiate secondary orchestration [41]. On a molecular level, key transcriptional activators circadian locomotor output cycles kaput (CLOCK), and brain muscle ARNT-like protein 1 (BMAL1), entrain circadian rhythms. Whereas intracellular CLOCK levels rarely fluctuate, BMAL1 increases in the morning, accompanying the binding of CLOCK and BMAL1 [42]. This heterodimerization of CLOCK and BMAL1 leads to the transcription of other clock genes including period circadian protein homologue (*PER*) and cryptochrome (*CRY*) during the day [43,44]. At night, accumulated PER and CRY proteins form heterodimers, and translocate from the cytosol to the nucleus [44]. This complex then inhibits CLOCK-BMAL1 heterodimerization, and the resultant transcription of PER-CRY mediated by the CLOCK-BMAL1 complex is also terminated [43,44]. The SCN regulates circadian secretion of the pineal hormone melatonin [45,46]. As this pineal hormone crosses the placenta without any alteration, it freely enters the fetal circulation and conveys photoperiodic information to the fetus [13].

**Figure 1:**
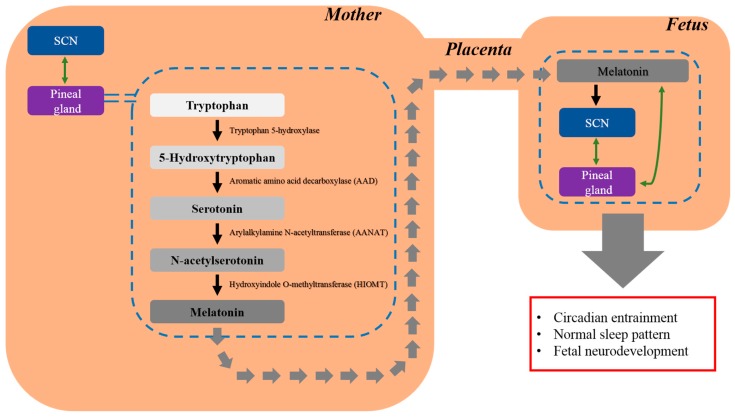
Maternal melatonin crosses the placental barrier to entrain the fetal circadian rhythm. Thus, melatonin is present in the fetal brain prior to the maturation of the fetal pineal gland. After crossing the placenta, melatonin entrains the fetal circadian rhythm, maintains the normal sleep pattern, and protects the fetus from neurodevelopmental disorders such as ASD.

Melatonin exerts its effects via two types of receptors: membrane-associated receptors (M1 and M2) [47] and nuclear receptors, which belong to the superfamily of retinoid orphan receptors (ROR) and the subfamily of retinoid z receptors (RZR) [48]. The members of the ROR/RZR subfamily include the splicing variants of RORα (RORα1, RORα2, RORα3, RZRα) [48]. Melatonin receptors are widespread in the fetus and have been identified in the suprachiasmatic nucleus and peripheral organs [13,26]. The MT1 melatonin receptor is expressed in human amniotic epithelial cells [49]. Activation of the MT1 receptor exerts a neuroprotective effect against oxidative stress, resulting in increased neuronal differentiation and survival rate [49]. Melatonin crosses the placenta and introduces the daily melatonin rhythm, which is characterized by high levels at night and low levels during the day, to the fetus [13]. Melatonin mediates organ functions according to the circadian cycle. Additionally, melatonin may play a crucial role in fetal neurodevelopment, because the normal sleep pattern, which is the circadian rhythm influenced by melatonin, is known to affect neurodevelopment [13,26]. In this regard, melatonin may play a variety of roles, rather than being confined to circadian entraining (Figure 1).

### 2.2. Regulatory Role of Melatonin in Fetal Development and Neuroprotection

As described above, the normal sleep pattern is an important factor in neurodevelopment [13,26]. The normal sleep pattern consists of two states: non-rapid eye movement (NREM), and rapid eye movement (REM) [50]. Studies have shown that neural development mainly occurs during the REM state [51]. In addition, human newborns sleep 16–18 h a day, and more than 50% of their sleep state is REM [52]. A newborn is likely to undergo vigorous neural development during REM sleep. In this context, the neurodevelopment of a fetus is disrupted if its REM sleep is disrupted [51]. REM sleep is closely associated with the pineal hormone melatonin. Melatonin extends the duration of the REM state, whereas a lack of this hormone increases NREM periods [26]. In a clinical study, Anderson et al. [53] found that a melatonin dose of 3 mg or less reduced sleep disorders in children with ASD. Furthermore, melatonin acts as a neuroprotectant for the fetus. The hormone has been shown to reduce the risk of cell death and inflammation in the fetal brain in an animal model of hypoxia [13,54] and to increase the survival rates in newborns with asphyxia by reducing oxidative stress [13,55]. In summary, melatonin affects rapid eye movement sleep, exerts a neuroprotective effect, and plays a role in neurodevelopment. The circadian rhythm of melatonin synthesis emerges 6 to 8 weeks after birth [56]. Circadian variation in melatonin levels are evident 3 to 6 months after birth, and the nocturnal peak of melatonin synthesis occurs between 4 and 7 years after birth [57]. These findings suggest that melatonin entrains circadian rhythms in the fetus. Additionally, altered levels of melatonin-synthesizing enzymes, which are heritable, may underlie abnormal melatonin synthesis [34,35]. The fact that the melatonin-synthesizing pathway is frequently impaired in individuals with ASD [33] suggests that melatonin plays a significant role in the development of ASD. Therefore, it is likely that melatonin is involved in the development of fetal circadian rhythms and ASD.

## 3. Melatonin and Its Implications for Autism Spectrum Disorder (ASD)

### 3.1. Overview of ASD

Autism comprises a series of disorders that vary in severity, intellectual level, and functional disability. The fifth revised version of the Diagnostic and Statistical Manual of Mental Disorders (DSM-5) combined specific diagnoses and suggested the single broad ASD diagnosis [58]. ASD refers to the range of neurodevelopmental disorders accompanying impairments in social interaction, difficulties in communication, and stereotyped or repetitive behaviors. Because the symptoms of autism vary enormously, the term “autism spectrum disorder” encompasses a single diagnostic category of autism involving numerous conditions [37]. Whereas the older term, autism, described a specific diagnostic category, the newer term, ASD, better explains this disorder by including multiple conditions [37]. In this regard, the older term is being replaced by the newer term ASD. Genetic disruption may give rise to synaptic deficits, and ultimately cause ASD. It has been revealed that ASD-related genes are involved in common signal transduction pathways that are responsible for synaptic development and neuronal plasticity [37].

### 3.2. Abnormal Melatonin Secretion and Its Implication in ASD

Melatonin was suggested as a potential therapeutic intervention for FXS with ASD in our previous review article. In the article, FXS was the most common form of ASD and seemed to be associated with the loss of fragile X mental retardation (fmr) gene products such as fragile X mental retardation protein (FMRP), leading to diverse physiological and behavioral abnormalities. Additionally, the mutation of this gene disrupts the normal sleep pattern and circadian rhythm. Subsequent alterations of melatonin synthesis and melatonin-dependent pathways may lead to autistic behaviors [37]. Melatonin is a well-known modulator of the regulation of neural plasticity and circadian rhythm [59,60]. Thus, abnormal melatonin levels may destroy the circadian rhythm, and may even result in autistic behavior. Studies have reported decreased melatonin concentrations in individuals with ASD. Reduced levels of serum melatonin were found in autistic patients [17]. Other studies have demonstrated similar trends. According to Kulman et al. [18] melatonin concentrations in autistic children are lower than those in normal children. They suggested that pineal hypofunction in autistic children may be the cause of these reduced melatonin levels. Other researchers have also reported decreased nocturnal melatonin production in autistic individuals [61]. Also, as mentioned above, neurodevelopment mainly occurs during normal sleep. Therefore, children with neurodevelopmental disorders including ASD may suffer from pediatric insomnia. For these patients, melatonin may play a beneficial role not only as a neuroprotectant but also as a circadian entrainer [62]. In this context, abnormalities in melatonin concentration are likely to increase the risk of ASD.

Melatonin is known to freely cross the placental barrier [63]. Even before the maturation of the pineal gland, which is responsible for melatonin secretion, melatonin can be detected in the fetal brain. Melatonin defends against neonatal inflammation and brain injury, evidenced by reduced post-inflammatory unfolded protein response (UPR) and normalization of autophagy following melatonin treatment [64]. Maternal and placental melatonin contribute to fetal neurodevelopment [65]. Thus, abnormalities in maternal melatonin levels may be linked to an augmented risk of fetal neurodevelopmental disorders [66]. Additionally, abnormal maternal melatonin may cause excessive oxidative stress [65]. As the central nervous system consumes a great deal of energy, has few endogenous antioxidants, including catalase and superoxide dismutase, and undergoes vigorous cell differentiation and proliferation, it is highly susceptible to oxidative stress [65]. Therefore, the antioxidant role of melatonin is vital for normal neurodevelopment, especially in the fetus.

**Figure 2:**
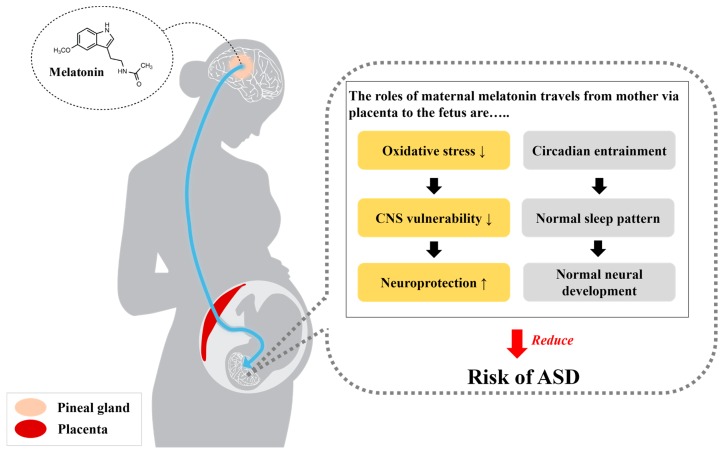
The beneficial roles of maternal melatonin that travels from mother via placenta to the fetus. The functions of melatonin in neuroprotection and circadian entraining may reduce the risk of ASD. Normal melatonin concentrations during pregnancy contribute to neuroprotection and the normal neurodevelopment of the fetus through the inhibition of excessive oxidative stress in the vulnerable central nervous system. Additionally, as adequate melatonin levels maintain the normal sleep pattern and circadian rhythm, normal melatonin secretion may also elicit neurodevelopment.

Thus, mainly as a neuroprotectant, circadian entrainer, and antioxidant, melatonin is thought to protect the fetus from neurodevelopmental disorders and to relieve abnormal oxidative stress, and may reduce the risk of ASD (Figure 2).

## 4. Conclusions and Perspectives

The properties of melatonin have been reported by a number of researchers. As described above, this hormone plays multiple roles, including neuroprotection and circadian entraining. Normal melatonin concentrations during pregnancy aid in neuroprotection and normal neurodevelopment of the fetus through the inhibition of excessive oxidative stress in the vulnerable central nervous system. Additionally, as the normal sleep pattern and circadian rhythm are maintained by sufficient melatonin levels, normal melatonin secretion may also influence neurodevelopment. Eventually, the well-known functions of melatonin in neuroprotection and circadian entraining may reduce the risk of ASD. Moreover, the prevalence of circadian disturbances in individuals with ASD suggests that circadian rhythm may a predictive indicator of ASD.
